# Bone Morphogenetic Protein 13 Has Protumorigenic Effects on Hepatocellular Carcinoma Cells In Vitro

**DOI:** 10.3390/ijms241311059

**Published:** 2023-07-04

**Authors:** Vanessa Kersten, Tatjana Seitz, Judith Sommer, Wolfgang E. Thasler, Anja Bosserhoff, Claus Hellerbrand

**Affiliations:** 1 Institute of Biochemistry, Friedrich-Alexander-University Erlangen-Nürnberg, D-91054 Erlangen, Germany; vanessa.peschl@fau.de (V.K.); tatjana.seitz@fau.de (T.S.); judith.sommer@fau.de (J.S.); anja.bosserhoff@fau.de (A.B.); 2 Human Tissue and Cell Research-Services GmbH, Am Klopferspitz 19, D-82152 Planegg, Germany; wolfgang.thasler@swmbrk.de; 3 Comprehensive Cancer Center (CCC) Erlangen-EMN, D-91054 Erlangen, Germany

**Keywords:** BMP13, hepatocellular carcinoma, hepatic stellate cells

## Abstract

Activated hepatic stellate cells (HSCs) play a key role in hepatic fibrosis and, thus, build the “soil” for hepatocarcinogenesis. Furthermore, HSCs are known to promote the progression of hepatocellular carcinoma (HCC), but the molecular mechanisms are only incompletely understood. Recently, we newly described the expression of bone morphogenetic protein 13 (BMP13) by HSCs in fibrotic liver tissue. In addition, BMP13 has mostly been studied in the context of cartilage and bone repair, but not in liver disease or cancer. Thus, we aimed to analyze the expression and function of BMP13 in HCC. Expression analyses revealed high BMP13-expression in activated human HSCs, but not in human HCC-cell-lines. Furthermore, analysis of human HCC tissues showed a significant correlation between BMP13 and α-smooth muscle actin (α-SMA), and immunofluorescence staining confirmed the co-localization of BMP13 and α-SMA, indicating activated HSCs as the cellular source of BMP13 in HCC. Stimulation of HCC cells with recombinant BMP13 increased the expression of the *inhibitors of differentiation 1* (*ID1*) and *2* (*ID2*), which are known targets of BMP-signaling and cell-cycle promotors. In line with this, BMP13-stimulation caused an induced SMAD 1/5/9 and extracellular signal-regulated kinase (ERK) phosphorylation, as well as reduced expression of *cyclin-dependent kinase inhibitors 1A* (*CDKN1A*) and *2A* (*CDKN2A*). Furthermore, stimulation with recombinant BMP13 led to increased proliferation and colony size formation of HCC cells in clonogenicity assays. The protumorigenic effects of BMP13 on HCC cells were almost completely abrogated by the small molecule dorsomorphin 1 (DMH1), which selectively blocks the intracellular kinase domain of ALK2 and ALK3, indicating that BMP13 acts via these BMP type I receptors on HCC cells. In summary, this study newly identifies stroma-derived BMP13 as a potential new tumor promotor in HCC and indicates this secreted growth-factor as a possible novel therapeutic target in HCC.

## 1. Introduction

Hepatocellular carcinoma (HCC) is one of the most aggressive tumors. It is the third-leading cause of cancer-related deaths worldwide, with its incidence still rising [1,2].

Although surgery is the primary curative treatment, in most cases, HCC is diagnosed in already-advanced stages, with lack of effective treatment options [3]. Over the past decade, novel systemic treatment options for patients with advanced HCC have evolved. In addition to the multi-tyrosine kinase inhibitor sorafenib, other targeted agents and immune checkpoint inhibitors have been shown to be associated with improved survival in subsets of patients. Nevertheless, severe side effects and the rapid development of resistance are major clinical hurdles. Therefore, the identification of novel therapeutic targets and diagnostic markers is urgently needed [4].

Liver fibrosis and cirrhosis are major risk factors for developing liver cancer [5]. The activation of hepatic stellate cells (HSCs) is the key event of hepatic fibrosis [6] and, thus, HSCs form the “soil” for hepatocarcinogenesis. Moreover, activated HSCs/myofibroblasts form and permeate the HCC stroma, and there is strong evidence that stromal HSCs promote HCC progression [7,8]. However, the molecular mechanisms by which HSCs act on HCC cells are only incompletely understood.

Bone morphogenetic proteins (BMPs) form a group of multifunctional secreted cytokines that belong to the transforming growth factor β (TGFβ) superfamily. To date, more than 15 BMPs with highly conserved structures have been identified in vertebrates. They play a critical role in various cell processes, such as tissue development, cell differentiation, proliferation, and apoptosis [9]. BMP dysregulation is associated with the development and progression of different diseases, including various cancers [10]. Nevertheless, our current knowledge of BMPs in cancer is far from clear [11]. It has been shown that some BMPs, such as BMP4 and BMP9, act as protumorigenic factors in HCC [12,13]. On the other hand, antitumorigenic effects have been described for other BMPs, such as BMP2 and BMP7 in HCC [14,15].

BMP13 (also known as growth differentiation factor 6) is a relatively new and insufficiently studied BMP, compared to most other BMP family members. So far, it has been mostly studied in the context of cartilage and bone repair in vitro and in vivo [16,17,18]. In 2018, a study first analyzed BMP13 in the context of cancer. Here, it was shown that BMP13 is upregulated in melanoma and that BMP13 inhibits cell death and promotes the growth of melanoma cells [19]. Furthermore, the expression of BMP13 in chronic liver disease was previously unknown. Recently, we found that BMP13 is upregulated in liver fibrosis, and we identified activated HSCs as the cellular source of hepatic BMP13 in fibrotic liver tissue [20].

## 2. Results

### 2.1. BMP13 Expression in Hepatocellular Carcinoma

**Figure 1:**
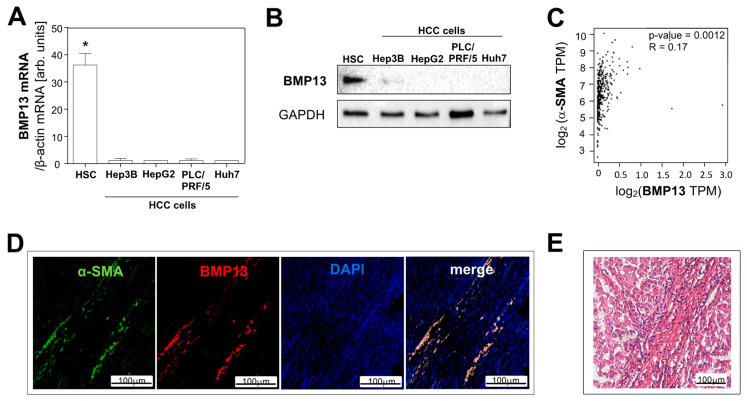
Expression of BMP13 in hepatocellular carcinoma (HCC). (**A**) Analysis of mRNA expression of *BMP13* in activated hepatic stellate cells (HSCs) and four human HCC cell lines. (**B**) Western blot analysis of BMP13 protein expression in HSCs and four human HCC cell lines. GAPDH was used as a housekeeper. Densitometric analysis (BMP13/GAPDH) revealed that, compared to the BMP13 signal in HSCs, the immunosignal in HCC cells was 20 to 210-fold lower (20-fold in Hep3B cells, 23-fold in HepG2 cells, 210-fold in PLC/PRF/5 cells, and 30-fold in Huh7 cells). (**C**) Correlation of BMP13 and α-smooth muscle actin (α-SMA) RNA expression levels (log_2_(transcript per million)) in 361 human HCC tissues. The cancer genome atlas (TCGA)-derived data were used, applying the gene expression profiling interactive analysis (GEPIA) database. (**D**) Representative image of immunofluorescence staining for BMP13 (red) and α-SMA (green) in human HCC tissue section. (**E**) Hematoxylin and eosin staining of the same tissue section area as shown in panel (**D**) (*: *p*  <  0.05).

We recently identified activated HSCs as the cellular source of BMP13 in fibrotic liver tissue [20]. Therefore, we first compared the expression of BMP13 in activated human HSCs and four different human HCC cell lines. RT-qPCR analysis revealed that *BMP13* mRNA expression is more than 30-fold higher in HSCs than in HCC cells (Figure 1A). Western blot analysis confirmed BMP13 expression in activated HSCs but no signal was detectable in HCC cells (Figure 1B). In silico analysis of 361 human HCC tissue samples, applying the GEPIA database [21], showed a significant correlation between the expression of BMP13 and α-smooth muscle actin (α-SMA), a specific marker for activated HSCs [6,22] (Figure 1C). No correlation was found between the expression of BMP13 and CYP7A1, which is a marker for hepatocytes, or between the expression of BMP13 and the epithelial cell adhesion molecule (EpCAM), which has been shown to be produced by HCC cells (Figure S1A,B) [23,24]. Furthermore, immunofluorescence analysis of human HCC tissue showed colocalization of BMP13 and α-SMA (Figure 1D and Figure S1C). In summary, these data indicate that activated HSCs might be not only the cellular source of BMP13 in fibrotic/cirrhotic liver tissue, but also in HCC.

### 2.2. Effect of BMP13 on Tumorigenicity of HCC Cells

**Figure 2:**
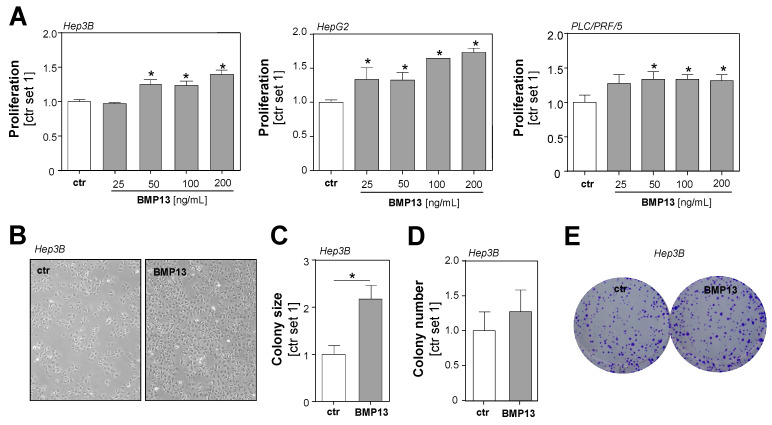
Effect of BMP13 on tumorigenicity of HCC cells in vitro. (**A**) Analysis of the proliferation of HCC cell lines Hep3B, HepG2, and PLC/PRF/5 after 24 h stimulation with rBMP13. The proliferation rate of control cells has been set as 1 and the effect of BMP13 is shown as the relative induction of proliferation in stimulated cells, compared to control cells. (**B**) Representative images of Hep3B cells stimulated without (ctr) and with recombinant BMP13 for 24 h (40-fold magnification). Quantification of (**C**) colony size, (**D**) colony number, and (**E**) representative images (1-fold magnification) in anchorage-dependent clonogenic assays with Hep3B treated without (ctr) or with rBMP13 for 9 days. The size and number of colonies of control cells have been set as 1 and the effect of BMP13 is shown as relative induction compared to control cells (*: *p*  <  0.05).

Because BMP13 is a secreted protein, to obtain a first insight into the effect of BMP13 in HCC, we stimulated human HCC cells with recombinant BMP13 (rBMP13) and performed functional in vitro assays. Stimulation with rBMP13 for 24 h induced the proliferation of HCC cells in a dose-dependent manner (Figure 2A,B). Furthermore, we assessed the impact of BMP13 on HCC cells in clonogenicity assays, which also reflected stem cell properties and cell survival of tumor cells. Here, treatment with rBMP13 was associated with an increase in colony size in HCC cells (Figure 2C and Figure S2A); in addition, the colony number was slightly higher in BMP13-treated HCC cells, although the difference to controls did not reach the level of significance (Figure 2D,E and Figure S2B,C).

**Figure 3:**
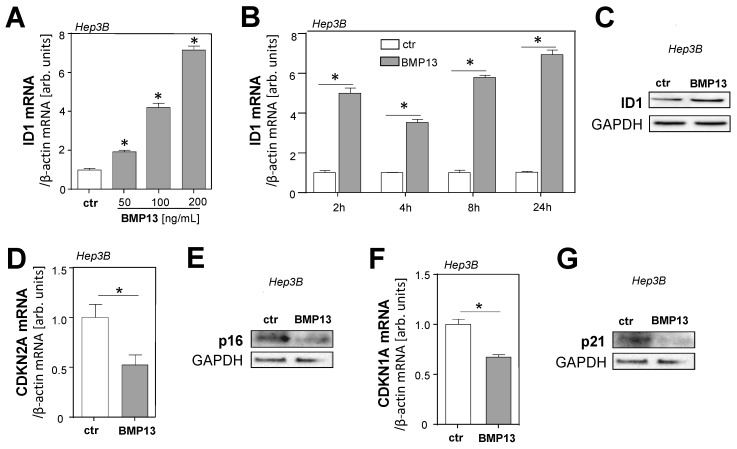
Effect of BMP13 on ID1 and cell-cycle regulators in HCC cells. (**A**) Analysis of *ID1* mRNA expression in Hep3B cells treated with different doses of recombinant BMP13 (rBMP13) for 2 h. (**B**) Analysis of mRNA expression of *ID1* in Hep3B cells after treatment with rBMP13 (200 ng/mL) for different time points. (**C**) Western blot analysis of ID1 expression in rBMP13 (200 ng/mL) treated and Hep3B control cells (ctr) after 24 h. GAPDH was used as a housekeeper. Densitometric analysis (ID1/GAPDH) confirmed an increased (1.9-fold) staining signal of ID1 in BMP13-treated cells. (**D**) Analysis of *CDKN2A* mRNA expression in Hep3B stimulated without (ctr) or with rBMP13 (200 ng/mL) for 24 h. (**E**) Western blot analysis of p16 protein expression in Hep3B cells stimulated with rBMP13 for 24 h. GAPDH was used as a housekeeper. Densitometric analysis (p16/GAPDH) showed a decreased (0.8-fold) staining signal of p16 in BMP13 treated cells. (**F**) Analysis of *CDKN1A* mRNA expression in Hep3B stimulated without (ctr) or with rBMP13 (200 ng/mL) for 24 h. (**G**) Western blot analysis of p21 protein expression in Hep3B cells stimulated with rBMP13 for 24 h. GAPDH was used as a housekeeper. Densitometric analysis (p21/GAPDH) showed a decreased (0.4-fold) staining signal of p21 in BMP13-treated cells (*: *p*  <  0.05).

The transcription factor inhibitor of differentiation 1 (ID1) has been identified as a direct BMP target gene [25], and the high expression of ID1 has been shown to promote HCC progression via inducing growth and metastatic ability [26,27]. Therefore, we analyzed the effects of rBMP13 on ID1 expression in HCC cells. Stimulation with rBMP13 caused the induction of *ID1* mRNA expression in HCC cell lines in a dose- and time-dependent manner (Figure 3A,B and Figure S3A,B). Western blot analysis confirmed increased ID1 protein levels in HCC cells following stimulation with rBMP13 (Figure 3C and Figure S3C). In addition to ID1, the inhibitor of differentiation 2 (ID2) has also been shown to be induced by BMP stimulation and, furthermore, ID2 has been shown to induce cell cycle progression in HCC [28]. Thus, we also analyzed the effect of rBMP13 on the expression of this transcription factor and found that stimulation with rBMP13 caused a dose- and time-dependent induction of *ID2* mRNA in HCC cells (Figure S3D,E). As it has been previously described that ID1 can modulate the expression of cyclin-dependent kinase inhibitors (CDKNs) in HepG2 cells [29], we investigated the effect of BMP13 on *CDKN1A* (p21) and *CDKN2A* (p16) expression in HCC cells. Stimulation with BMP13 significantly reduced the expression of p16 in HCC cells (Figure 3D,E and Figure S3F,G). Furthermore, p21 was significantly repressed by BMP13 in Hep3B cells (Figure 3F,G), but only slightly downregulated in PLC/PRF/5 und HepG2 cells (Figure S3H,I).

Taken together, these data identify ID1 and ID2 as new target genes of BMP13 in HCC cells and indicate that BMP13-induced proliferation of HCC cells might be mediated by the upregulation of ID1 and, potentially, ID2, and by the subsequent suppression of CDKNs.

### 2.3. Molecular Mechanisms of BMP13 Effects on HCC Cells In Vitro

**Figure 4:**
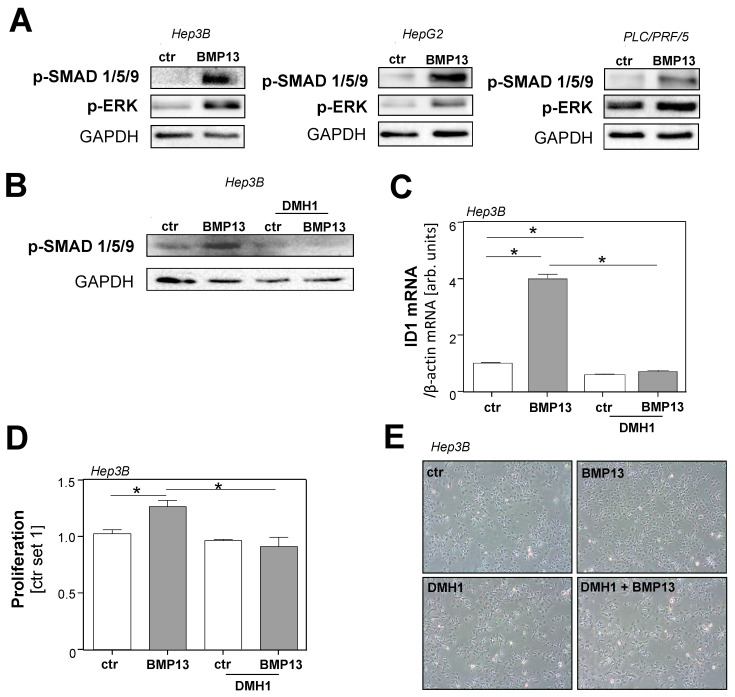
Effect of BMP receptor inhibitor dorsomorphin 1 (DMH1), which inhibits ALK2 and ALK3 in HCC cells. (**A**) Western blot analysis of phosphorylated SMAD 1/5/9 and phosphorylated ERK in HCC cells after stimulation with rBMP13 (200 ng/mL) for 20 min and control cells (ctr). GAPDH was used as a housekeeper. Densitometric analysis (p-SMAD 1/5/9 /GAPDH) confirmed an increased staining signal of p-SMAD 1/5/9 in Hep3B cells (18.7-fold), HepG2 cells (5.9-fold), and PLC/PRF/5 cells (4.8-fold) treated with rBMP13. Densitometric analysis (p-ERK/GAPDH) confirmed an increased staining signal of p-ERK in Hep3B cells (4.4-fold), HepG2 cells (2.9-fold), and PLC/PRF/5 cells (2.0-fold) treated with rBMP13. (**B**) Effect of BMP receptor inhibitor DMH1 (10 nM) in Hep3B cells on rBMP13 (200 ng/mL)-induced phosphorylation of SMAD 1/5/9. Cells were preincubated with DMH1 for 15 min before 15 min stimulation with rBMP13. Densitometric analysis (p-SMAD 1/5/9 /GAPDH) confirmed that the p-SMAD 1/5/9 signal induced by BMP13 (2.1-fold compared to control) was completely abolished (0.45 compared to control). (**C**) Effect of DMH1 (10 nM) on rBMP13 (200 ng/mL)-induced *ID1* mRNA expression. For mRNA expression analysis, cells were treated with DMH1 for 20 min and then stimulated with rBMP13 for 8 h. Effect of BMP receptor inhibitor DMH1 (10 nM) in Hep3B cells on rBMP13 (200 ng/mL)-induced (**D**) proliferation of Hep3B (**E**) and representative images (40-fold magnification) of Hep3B cells. Proliferation was assessed after 24 h stimulation with rBMP13. Proliferation of control cells was set as 1 and the effect of BMP13 is shown as relative induction of the proliferation rate compared to ctr-cells (*: *p*  <  0.05).

Next, we analyzed the effect of stimulation with recombinant BMP13 on the phosphorylation of SMAD 1/5/9 in HCC cells in vitro. Here, we observed a strong induction in all three human HCC cell lines (Figure 4A).

It has been stated that, particularly in cancer cells, BMPs frequently also activate SMAD 2/3 signaling [30]. Nevertheless, we did not observe SMAD 2 phosphorylation after stimulation with BMP13 in HCC cells (Figure S4).

In addition to SMAD signaling, BMPs have been shown to induce the activation of mitogen-activated protein kinase (MAPK), such as extracellular-regulated kinase (ERK), which is known to induce the tumorigenicity of HCC cells [31]. Interestingly, we found that the phosphorylation levels of ERK increased significantly after stimulating HCC cells with BMP13 (Figure 4A).

Next, we aimed to identify the receptors that mediate the protumorigenic effects of BMP13 on HCC cells. Here, we focused on ALK1, ALK2, ALK3, and ALK6, as it has been shown that the activation of these BMP type I receptors can lead to the phosphorylation of SMAD 1/5/9 [32]. Dorsomorphin 1 (DMH1) is a small selective BMP receptor inhibitor which effectively blocks the intracellular kinase domain of ALK2 and ALK3 [33]. DMH1 completely blocked the rBMP13-induced SMAD 1/5/9 phosphorylation in HCC cells (Figure 4B and Figure S5A). Furthermore, DMH1 significantly reduced both basal and BMP13-induced *ID1* and *ID2* induction in HCC cells (Figure 4C and Figure S5B,C). Moreover, DMH1 completely inhibited the growth-inducing effect of BMP13 on Hep3B cells (Figure 4D,E).

In summary, these data indicate that the induction of the canonical BMP-signaling pathway and the growth-promoting effect of BMP13 on HCC cells is mediated through binding to ALK2 and/or ALK3.

## 3. Discussion

The aim of this study was to obtain insight into the role of BMP13 in HCC. To date, BMP13 has mostly been studied in cartilage and tendon healing processes; only one study analyzed this BMP-family member in the context of cancer. Venkatesan et al. stated that BMP13 promotes the progression of melanoma by inducing proliferation and inhibiting the apoptosis of melanoma cells [19].

Previous studies of other BMP-family members in HCC have shown that the effects of these growth factors can be diverse. For example, BMP7 mRNA expression was significantly downregulated in tumor tissues, compared with corresponding non-tumorous tissues. In addition, the downregulation of SMAD 1/5/9 was observed in carcinoma tissues, indicating that the BMP7/SMAD signaling pathway may act as a self-defense mechanism against carcinogenesis and the progression of HCC [14]. BMP2 was also able to inhibit the proliferation of HCC cells in a dose-dependent manner. In a complementary approach, knockdown of BMP2 resulted in enhanced proliferation and migration of HCC cells. Overexpression of BMP2 induced a rapid increase in p21 expression, which led to G1 arrest [15]. In contrast, BMP9 promotes the growth of liver cancer cell lines [12]. Furthermore, knockdown of BMP4 led to inhibition of clonogenic activity, migration, and the invasive potential of HCC [13].

In this study, we provide the first evidence showing that BMP13 acts as a protumorigenic factor in HCC. We found that recombinant BMP13 induces proliferation and colony formation in different human HCC cell lines in vitro. Furthermore, we found that recombinant BMP13 induces the expression of *ID1* and *ID2* in a dose-dependent manner in HCC cells, and that BMP13 represses the expression of *CDKN1A* (p21) and *CDKN2A* (p16) in HCC cells. ID1 and ID2 have been shown to induce the proliferation of HCC cells [28,34]. Furthermore, it has been shown that ID1 has an inhibitory effect on p16 in HCC cells [34]. Thus, it appears likely that at least part of the growth-inducing effect of BMP13 on HCC cells is mediated via ID1 (and potentially also by ID2 induction) and a stimulating effect on the cell cycle, respectively. However, further functional in vitro and in vivo analysis need to be performed to confirm this connection.

As the main cellular source of BMP13 in HCC, we determined that activated HSCs and the small-molecule BMP receptor inhibitor DMH1 effectively blocked the observed effects of recombinant BMP13 on HCC cells in vitro. These findings suggest that BMP13 mainly acts through ALK2 and/or ALK3 on HCC cells. Furthermore, we recently indicated that BMP13 expression is also upregulated during hepatic fibrosis [20]. In addition, in fibrotic liver tissue, activated HSCs are the cellular source of BMP13, and we found that BMP13 exhibits profibrogenic effects on HSCs [20]. Further in vivo analyses are necessary to validate the role of BMP13 in chronic liver disease. However, considering the close pathological connection between liver fibrosis and liver cancer, the findings of the present study suggest that BMP13 is an attractive novel target to fight both liver fibrosis and liver cancer formation and progression. In addition to the inhibition of the BMP13 receptors, RNA interference technology might be used for targeted BMP13 suppression in the liver. It has been shown that intravenous siRNA application can be used for efficient gene depletion in HSCs [35]. Furthermore, targeted gene depletion in the liver with siRNA has previously been successfully tested in clinical trials and approved for the treatment of some types of liver disease [35,36].

In summary, this study newly describes BMP13 expression in HCC and provides the first evidence that stroma-derived BMP13 might act as a tumor promotor in HCC. Future studies are needed to validate the role of BMP13 and its molecular mechanisms of action in vivo and to elucidate the potential of this soluble growth factor as a diagnostic marker or anti-tumorigenic therapeutic target in patients with chronic liver disease.

## 4. Materials and Methods

### 4.1. Cells and Cell Culture

Primary human hepatic stellate cells (HSCs) were isolated and cultured, as described previously [37]. In vitro activation of HSCs was achieved by cell culture on uncoated tissue culture dishes [37]. Human liver tissue for cell isolation was obtained from the charitable, state-controlled Human Tissue and Cell Research (HTCR) foundation with patients’ informed consent [38]. The sampling and handling of patient materials were carried out according to the ethical principles of the Declaration of Helsinki. The HCC cell lines PLC/PRF/5 (ATCC CRL-8024), Hep3B (ATCC HB-8064), HepG2 (ATCC HB-8065), and Huh7 (ATCC PTA-4583) were cultured, as previously described [8].

For stimulation experiments, the cells were treated with recombinant human BMP13 (Biozol, Munich, Germany), TGFβ (Abcam, Cambridge, UK), or DMH1 (selective BMP receptor inhibitor, Sigma–Aldrich Chemie GmbH, Taufkirchen, Germany) in the concentrations indicated in the figures/results.

### 4.2. (Immuno)Histological Analysis

HCC tissue samples from cirrhotic patients were provided for immunohistological analyses by the charitable, state-controlled Human Tissue and Cell Research (HTCR) foundation, with patients’ informed consent [38].

For immunochemistry, HCC tissues were fixed in 4% PFA for 24 h and then deparaffinized, rehydrated, and boiled in a 0.01 M sodium citrate buffer solution (pH 8.0) for 40 min in a 90 °C water bath. After cooling to room temperature, slides for immunofluorescence staining were washed with PBS and permeabilized with 0.5% Triton X 100/1% PBS for 1 h. After that, tissues were incubated with anti-BMP13 antibodies (HPA045206, 1:400, Atlas Antibodies, SE-168 69, Bromma, Sweden) and anti-α smooth muscle actin antibodies (NB300-978SS, 1:500, Bio-Techne GmbH, Wiesbaden Nordenstadt, Germany) overnight at 4 °C and washed two times with PBS. Following incubation with an Alexa Fluor 488-conjugated donkey anti goat IgG (A11055, 1:1000, Invitrogen, Thermo Fisher Scientific, Waltham, MA, USA) and Cy3-conjugated donkey anti-rabbit IgG (711-165-152, 1:1000; Jackson ImmunoResearch Laboratories, Inc., West Grove, PA, USA) secondary antibody at room temperature for 1 h, sections were washed three times with PBS and mounted with DAPI (1:10,000).

For hematoxylin/eosin staining, paraffin embedded tissue sections were deparaffinized with Rotihistol for 1 min. Subsequently, tissue was rehydrated with a decreasing ethanol gradient and incubated with an aqueous solution of hematoxylin for 10 min. Tissue was washed with tap water until the slide was clear and counterstained for 1 min in an alcoholic solution of eosin, following a washing step.

Images were taken with an Olympus IX83 microscope with the ALTRA 20 Soft Imaging System^TM^ and CellSens Dimension Software version 2.3 (Olympus Soft Imaging Solutions GmbH, Münster, Germany) [39].

### 4.3. Analysis of mRNA Expression

For RT-qPCR, cellular RNA was isolated from cultured cells using the PureLink RNA Mini Kit (Thermo, Carlsbad, CA, USA). Subsequently, cDNA was generated by reverse transcriptase reaction. The RT-reaction was performed in 20 µL reaction volume containing 0.5 µg of total cellular RNA. RNAs were incubated in a GeneAmp^®^ PCR cycler (Applied Biosystems, Foster City, CA, USA) at 25 °C for 10 min, then for 15 min at 50 °C. Denaturation was performed at 85 °C for 5 min. cDNAs were controlled by PCR amplification of β-actin. Quantitative real-time PCR was performed using the LightCycler 480 System (Roche Diagnostics, Mannheim, Germany) [40] and the following sets of specific primers: β-actin human (forward: 5′-CTA CGT CGC CCT GGA CTT CGA GC-3′, reverse: 5′-GAT GGA GCC GCC GAT CCA CAC GG-3′), BMP13 human (forward: 5′-CAC CGT TGA CGC ATC TTG A-3′, reverse: 5′-CAA TGA AGG CAG AGA CCT GG-3′), ID1 human (forward: 5′-GTA TCT GCT TCG GGC TTC CA-3′, reverse: 5′-TGA TTC TTG GCG ACT GGC T-3′), ID2 human (forward: 5′-CAC GGA TAT CAG CAT CCT GTC CT-3′, reverse: 5′-CAA GTA AGA GAA CAC CCT GGG AAG A-3′, p16 human (forward: 5′-GCG GAA GGT CCC TCA GAC ATC CCC-3′, reverse: 5′-CTC GCA AGA AAT GCC CAC ATG AAT GTG C-3′) p21 human (forward: 5′-CGA GGC ACC GAG GCA CTC AGA GG-3′, reverse: 5′-CCT GCC TCC TCC CAA CTC ATC CC-3′).

### 4.4. Protein Analysis

For protein extraction, the cells were seeded in 6-well plates and incubated, and protein was isolated with lyse puffer (M-PER^TM^ Tissue Protein Extraction Reagent, Thermo, Rockford, IL, USA) with protease und phosphatase inhibitor (Halt^TM^ Protease und Phosphatase Inhibitor Single Use Cocktail, EDTA Free (100X), Thermo, Rockford, IL, USA). After blocking with 5% milk in PBS and washing three times, the following primary antibodies were applied overnight at 4 °C: rabbit anti-BMP13 (HPA045206, 1:1000, Atlas Antibodies, SE-168 69 Bromma, Sweden), mouse anti-ID1 (#sc1331-04, 1:500, Santa Cruz Biotechnology, Heidelberg, Germany), rabbit anti-phospho-p44/42 MAPK (Erk1/2) (Thr202/Tyr204) (#9101, 1:1000, Cell Signaling, Danvers, MA, USA) rabbit anti-phospho-SMAD 1/5/9 (#13820, 1:1000, Cell Signaling Technology), rabbit anti-phospho-SMAD 2 (#3101, 1:1000, Cell Signaling Technology), mouse anti-actin (MAB1501, 1:10,000; Merck Millipore, Billerica, MA, USA) and rabbit anti-GAPDH (#2118, 1:1000, Cell Signaling Technology). Blots were washed 3 times with PBS and secondary antibodies were incubated for 1 h at room temperature. Mouse anti-rabbit (sc-2357, 1:10,000, Santa Cruz Biotechnology), and anti-mouse (7076S, 1:3000, Cell Signaling Technology) were used as secondary antibodies [8]. Densinometric analysis was performed using the Image Lab Software version 5.2.1 (Bio-Rad Laboratories, Feldkirchen, Germany).

### 4.5. Analysis of Cell Proliferation

HCC cells were seeded into 96-well plates (4000 cells/well) in DMEM supplemented with 10% FCS and starved overnight. The next day, seeding cells were cultured in serum-free DMEM as control medium and the solution with the recombinant protein. Proliferation was measured after 24 h of stimulation. The proliferation of the cells was determined using a colometric XTT assay (Roche Diagnostics, Mannheim, Germany), according to the manufacturer’s protocol [8].

### 4.6. Clonogenic Assay

Clonogenic assays were used to analyze the ability of cancer cells to form colonies and the proliferation of cancer cells. The assays were based on the capability of single cells to grow into colonies, as described previously [41]. Briefly, HCC cells were seeded in 6-well plates (1000 cells/well) with 10% FCS. The next day, the cells were treated with recombinant BMP13 or serum-free DMEM as control medium. The cells were stimulated every third day. After 9 to 11 days, the cells were washed with PBS and fixed for 30 min with 300 µL 6% glutaraldehyde and 0.5% crystal violet. The plates were dried by room temperature, scanned and Olympus IX83 microscope with CellSens Dimension Software version 2.3 (Olympus Soft Imaging Solutions GmbH, Münster, Germany) was used to determine the number and size of the colonies in the 6-well plate.

### 4.7. Statistical Analysis

Calculations were performed by GraphPad Prism Software version 6.01 (GraphPad Software, San Diego, CA, USA). All analyses were performed in triplicate. Data are shown as the mean ± standard error of the mean (SEM). Data sets were compared with analysis of an unpaired Student’s *t*-test, one-way ANOVA, or two-way ANOVA, as appropriate, and a *p*-value < 0.05 was considered statistically significant.

## Data Availability

The data presented in this study are available on request from the corresponding author.

## Supplementary Materials

The following supporting information can be downloaded at: https://www.mdpi.com/article/10.3390/ijms241311059/s1.
