# Supplementary material for: Bone Morphogenetic Protein 13 Has Protumorigenic Effects on Hepatocellular Carcinoma Cells In Vitro
Source: Int J Mol Sci. 2023 Jul 4;24(13):11059. doi: 10.3390/ijms241311059 (PMC10342282; doi:10.3390/ijms241311059)
Supplement: Supplementary file 1 [file ijms-24-11059-s001.zip › ijms-2457965-supplementary.pdf]

Supplementary Figure S1

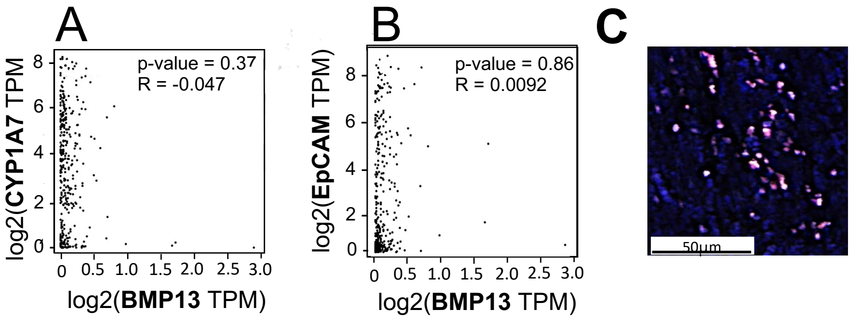

Figure S1. Expression of BMP13 in hepatocellular carcinoma (HCC). (**A**) Correlation of BMP13 and CYP7A1 RNA expression levels (log_2(transcript per million)) in 361 human HCC tissues. The cancer genome atlas (TCGA)-derived data were used applying the Gene Expression Profiling Interactive Analysis (GEPIA) database. (**B**) Correlation of BMP13 and epithelial cell adhesion molecule (EpCAM) RNA expression levels (log_2(transcript per million)) in 361 human HCC tissues. GEPIA database was used applying TCGA-derived data. (**C**) Representative image of immunofluorescence staining for BMP13 (red) and α-SMA (green) in human HCC tissue section.

Supplementary Figure S2

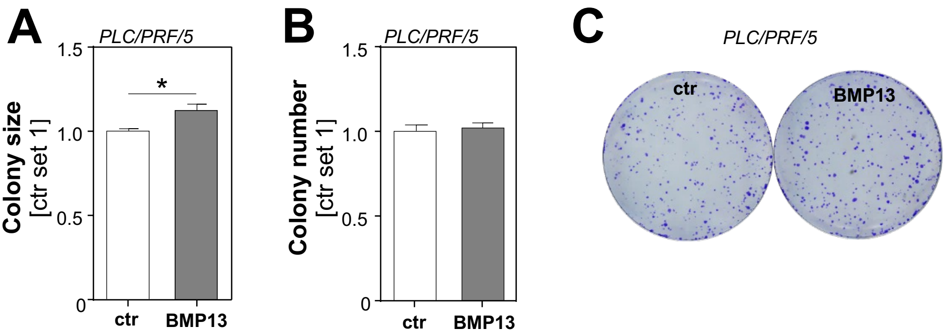

Figure S2. Effect of BMP13 on tumorigenicity of HCC cells in vitro. Quantification of (**A**) colony size, (**B**) colony number (**C**) and representative images (1-fold magnification) in anchorage-dependent clonogenic assays with PLC/PRF/5 treated without (ctr) or with rBMP13 for 11 days. Size and number of colonies of control cells have been set as 1 and the effect of BMP13 is shown as relative induction compared to control cells. (*: $p < 0.05$).

Supplementary Figure S3

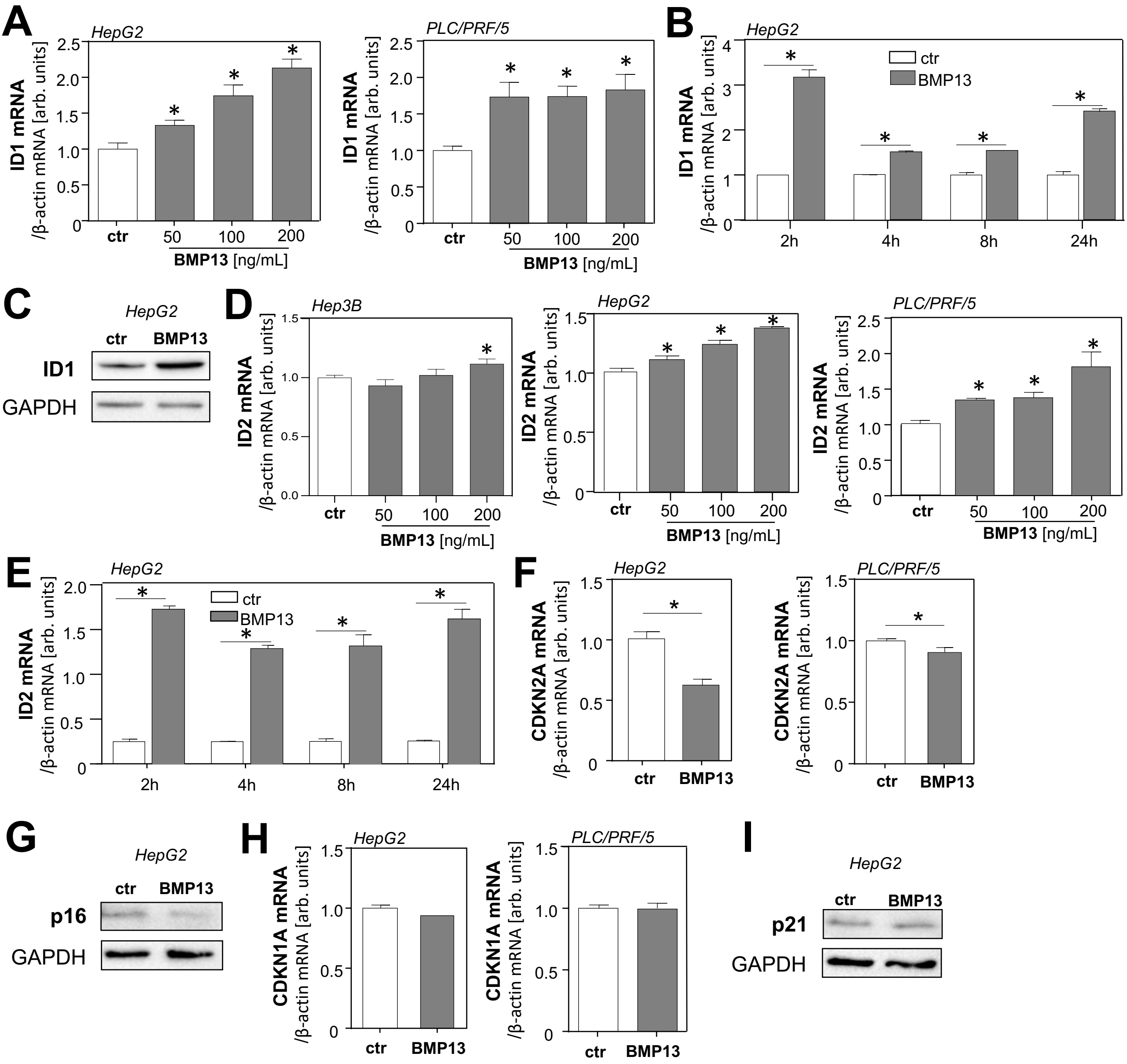

Figure S3. Effect of BMP13 on ID1, ID2 and cell-cycle regulators in HCC cells. (**A**) Analysis of *ID1* mRNA expression in HepG2 and PLC/PRF/5 cells treated with different doses of recombinant BMP13 (rBMP13) for 2 h. (**B**) Analysis of mRNA expression of *ID1* in HepG2 cells after treatment with rBMP13 (200 ng/mL) for different time points. (**C**) Western Blot analysis of ID1 expression in rBMP13 (200 ng/mL) treated and HepG2 control cells (ctr) after 24 h. GAPDH was used as a housekeeper. Densitometric analysis (ID1/GAPDH) showed an increased (2.6-fold) staining signal of ID1 in BMP13 treated cells. (**D**) Analysis of *ID2* mRNA expression in HCC cells treated with different doses of rBMP13 for 2 h. (**E**) Analysis of mRNA expression of *ID2* in HepG2 cells after treatment with rBMP13 (200 ng/mL) for different time points. (**F**) Analysis of *CDKN2A* mRNA expression in HepG2 and PLC/PRF/5 stimulated without (ctr) or with rBMP13 (200 ng/mL) for 24 h. (**G**) Western Blot analysis of p16 protein expression in HepG2 stimulated with rBMP13 for 24 h. GAPDH was used as a housekeeper. Densitometric analysis (p16/GAPDH) showed decreased (0.4-fold) staining signal of p16 in BMP13 treated HepG2. (**H**) Analysis of *CDKN1A* mRNA expression in HepG2 and PLC/PRF/5 stimulated without (ctr) or with rBMP13 (200 ng/mL) for 24 h. (**I**) Western Blot analysis of p21 protein expression in HepG2 stimulated with rBMP13 for 24 h. GAPDH was used as a housekeeper. Densitometric analysis (p21/GAPDH) showed no difference of p21 (1.05-fold) staining signal in rBMP13 treated HepG2 cells. (*: $p < 0.05$).

Supplementary Figure S4

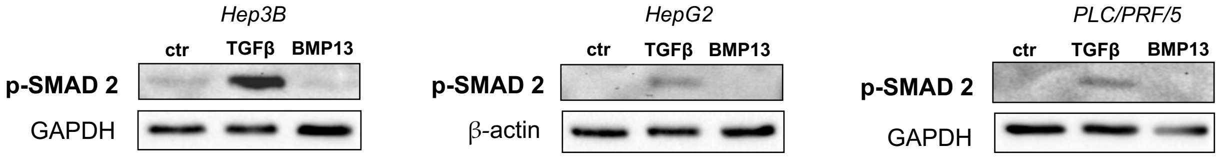

Figure S4. Effect of recombinant BMP13 on HCC cells in vitro. Western Blot analysis of phosphorylated SMAD 2 in recombinant BMP13 (200 ng/mL) treated and control cells (ctr). Recombinant transforming growth factor β (TGFβ) was used as positive control. GAPDH/β-actin were used as housekeepers. Densinometric analysis (p-SMAD 2/GAPDH) showed an increased (12.6-fold) staining signal of p-SMAD 2 in with recombinant TGFβ (rTGFβ) treated Hep3B but no difference (1.6-fold) staining signal in rBMP13 treated cells compared to control cells (ctr). Densinometric analysis (p-SMAD 2/GAPDH) showed an increased (4.4-fold) staining signal of p-SMAD 2 in rTGFβ treated HepG2 but no difference (0.5-fold) staining signal in rBMP13 treated cells compared to control cells. Densinometric analysis (p-SMAD 2/GAPDH) showed an increased (1.4-fold) staining signal of p-SMAD 2 in rTGFβ treated PLC/PRF/5 but no difference (1.1-fold) staining signal in rBMP13 treated cells compared to control cells. The cells were incubated for 20 minutes with the recombinant proteins.

Supplementary Figure S5

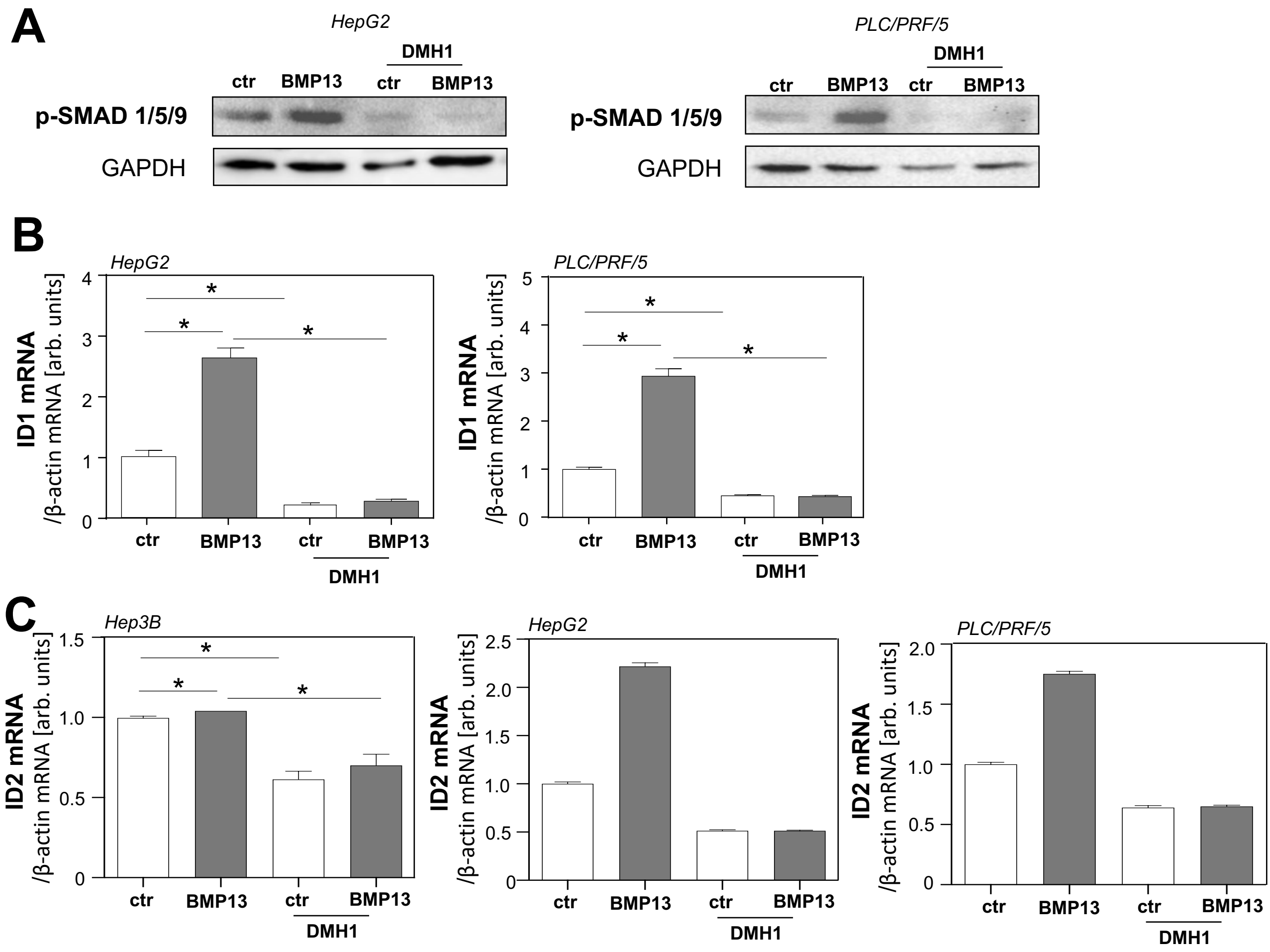

Figure S5. Effect of BMP receptor inhibitor dorsomorphin 1 (DMH1) which inhibits ALK2 and ALK3 in HCC cells. (**A**) Effect of BMP receptor inhibitor DMH1 (10 nM) in HepG2 and PLC/PRF/5 cells on rBMP13 (200 ng/mL)-induced phosphorylation of SMAD 1/5/9. Cells were preincubated with DMH1 for 15 minutes before 15 minutes stimulation with rBMP13. Densitometric analysis (p-SMAD 1/5/9 /GAPDH) confirmed that the p-SMAD 1/5/9 signal in HepG2 induced by BMP13 (1.8-fold compared to control) was completely abolished (1.0 compared to control). p-SMAD 1/5/9 signal in PLC/PRF/5 induced by BMP13 (2.6-fold compared to control) was also completely abolished (0.9 compared to control). Effect of DMH1 (10 nM) on rBMP13 (200 ng/mL)-induced (**B**) *ID1* mRNA expression (**C**) and *ID2* mRNA expression in HepG2 and PLC/PRF/5. For mRNA expression analysis cells were treated with DMH1 for 20 minutes and then stimulated with rBMP13 for 8 hours. (*: $p < 0.05$).
